# Supplementary material for: The development, implementation, and evaluation of an optimal model for the case detection, referral, and case management of Neglected Tropical Diseases
Source: PLoS One. 2023 May 10;18(5):e0283856. doi: 10.1371/journal.pone.0283856 (PMC10171595; doi:10.1371/journal.pone.0283856)

Optimal Model for the Case Detection, Referral and Confirmation of Neglected Tropical Diseases

GENDER EQUITY

- Gender balance factored in selection of CHWs and peer advocates
- Gender impact considered throughout implementation of case finding including gender of case finders and presence of family members

SUPERVISION

- Comprehensive supervision plan including tools, personnel and responsibilities at all levels
- Cascaded supervision structure to support skills transfer
- Supervision focus on the effective implementation of the training at all levels

INTEGRATED TRAINING

- Collaboratively developed integrated manual
- Peer advocate training Visual aids and context specific language
- Refresher training for CHWs based on visual aids
- Inclusion of integrated training manual in CHVs/CDDs MDA training
- Certificates for all training participants
- Cascaded Training Module for Community Health Workers and Peer Advocates

REFERRAL

- Referral tool adapted to include referee
- $5 USD per case identified for CHWs
- Two way Communication mechanism established (feedback/referral)

LOGISTICS

- Logistic toolkit for case detectors including flashlight batteries, T shirts and backpacks

NTD Case Finding
Optimal Model

An evidence based model for the effective active case identification and referral of Neglected Tropical Diseases in low resource settings

Integrated Training

- Cascaded Training Module for Community Health Workers and Peer Advocates
- Collaboratively developed integrated manual
- Visual aids and context specific language
- Refresher training for CHWs based on visual aids
- Inclusion of integrated training manual in CHVs/CDDs MDA training
- Peer advocate training
- Certificates for all training participants

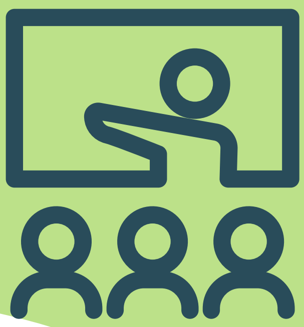

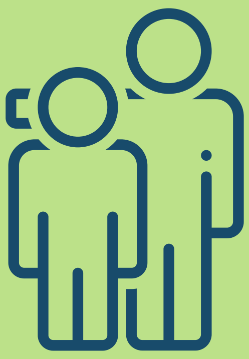

Supervision

- Comprehensive supervision plan including tools, personnel and responsibilities at all levels
- Cascaded supervision structure to support skills transfer
- Supervision focus on the effective implementation of the training at all levels;

Referral

- Referral tool adapted to include referee
- $5 USD per case identified for CHWs
- Two way Communication mechanism established (feedback/referral)

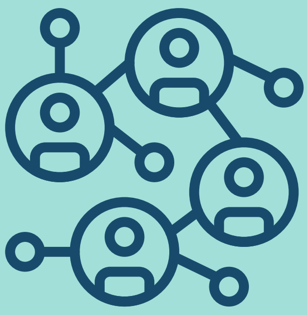

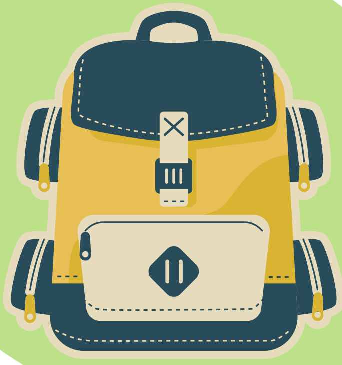

Logistics

- Logistic toolkit for case detectors including flashlight batteries, T shirts and backpacks

Gender Equity

- Gender balance factored in selection of CHWs and peer advocates
- Gender impact considered throughout implementation of case finding including gender of case finders and presence of family members

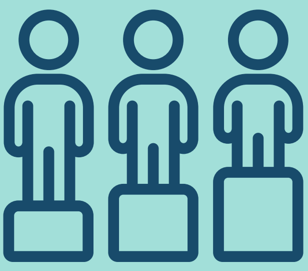

Supplement: S1 File — (PDF) [file pone.0283856.s001.pdf]
