# Supplementary figures and images for: The development, implementation, and evaluation of an optimal model for the case detection, referral, and case management of Neglected Tropical Diseases
Source: PLoS One. 2023 May 10;18(5):e0283856. doi: 10.1371/journal.pone.0283856 (PMC10171595; doi:10.1371/journal.pone.0283856)

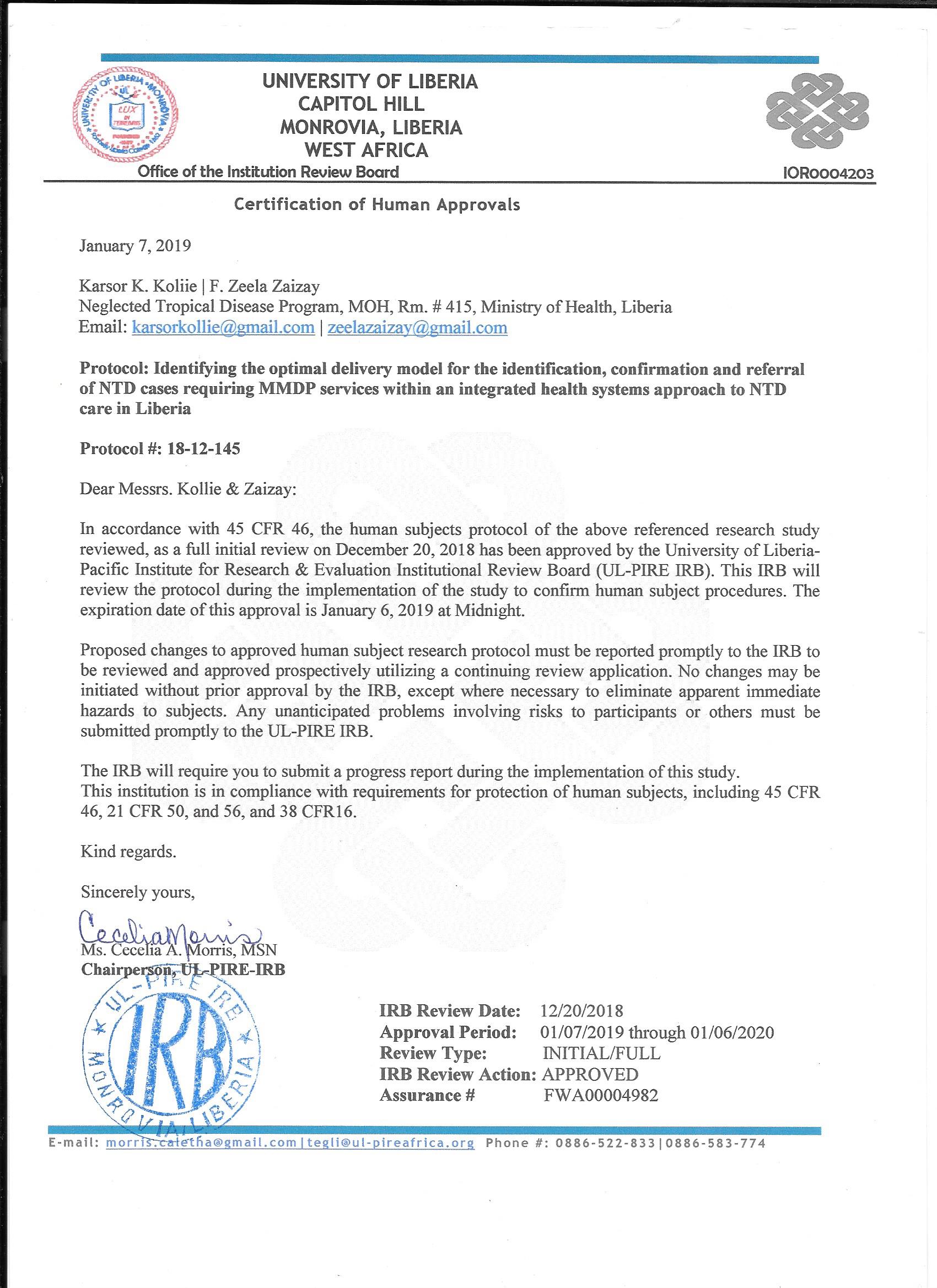

Supplement: S2 File — (TIFF) [file pone.0283856.s002.tiff]
