# Supplementary material for: The development, implementation, and evaluation of an optimal model for the case detection, referral, and case management of Neglected Tropical Diseases
Source: PLoS One. 2023 May 10;18(5):e0283856. doi: 10.1371/journal.pone.0283856 (PMC10171595; doi:10.1371/journal.pone.0283856)
Supplement: S3 File — (PDF) [file pone.0283856.s003.pdf]

Participant Information Sheets and Consent Forms

Participant Information Sheet for Focus Group Discussions with CHWs

Name of Principal Investigator: Karsor K. Kollie

Name of Organization: AIM Initiative, Ministry of Health Liberia NTD Programme, COUNTDOWN and Liverpool School of Tropical Medicine, funded by COR-NTD

Name of Project: Identifying the optimal delivery model for the identification, confirmation and referral of NTD cases requiring MMDP services within an integrated health systems approach to NTD care in Liberia

Introduction I am *(name)* from (*insert name of organisation*). I am doing research on diseases that affect the skin to try and improve how they are found and managed in the community by the health services in Liberia.

You are being invited to take part in a focus group discussion. Please take time to read the following information carefully and feel free to ask us if you would like more information or if there is anything that you do not understand. We would like to stress that you do not have to accept this invitation and should only agree to take part if you want to. Within receiving this information sheet, you have a day to decide whether you would like to take part. If so, you will be asked to sign a consent sheet.

Type of Discussion

The focus group discussions will be conducted with other people who have a similar job role as yourself and who are also willing to participate. This activity will last for **2 hours.** The aim of the discussion is to learn about and document your experiences using the new process for identification and referral of persons affected by skin diseases at the community level.

Participant Selection

You are being invited to participate in this research because of your experience as a health volunteer or worker who has been engaged in the identification and referral of people affected by skin diseases, which is important to us and the study.

Voluntary Participation

Your participation in this participatory workshop is entirely voluntary. It is your choice whether you want to participate or not.

Risks

If you feel uncomfortable at any point in the discussion, you do not have to take part if you don't wish to do so. If you would like to leave the discussion at any point, please just let the research team know.

Benefits

There will be no direct benefit to you, but your participation is likely to provide information on how to improve on integrated community-based case detection, referral and clinical diagnosis system for NTDs affecting the skin across a wide variety of contexts in Liberia.

Reimbursements

You will not be provided any incentive to take part in the research. However, we will reimburse any travel and provide refreshments during the workshops.

Confidentiality

If it is ok with you we would like to observe, take notes and audio record the focus group discussion. Once conducted, the information that we collect from these activities will be anonymised (your names will be removed) and stored securely in a confidential manner.

If in reporting the findings of this study your position as a person affected by skin disease makes you identifiable, we will discuss this with you prior to reporting these findings. We will do everything that we can to ensure confidentiality is maintained.

We encourage all participants to respect the privacy of others and not share what is said within the discussion with others who are not part of the discussions. We cannot guarantee this however so we encourage participants to only share what they feel comfortable to do so in a group situation.

Sharing the results

The knowledge obtained from this research will be shared with you before it is made available to the public. We will share the findings for the wider public so that other interested people may learn from the research. There is the possibility that the research project may be submitted for publication.

The notes and recordings from the sessions will be stored securely in password protected databases, which can only be accessed by designated researchers in country and abroad to the UK so that the information can be read and analysed by both teams. Audio recordings will be uploaded onto the secure electronic files and deleted from the recorders. The stored audio recordings will be deleted only after transcripts have been verified by an independent researcher, full analysis has taken place and results have been published. All other data collected will be stored securely, deleted and destroyed seven years after the study.

Right to Refuse or Withdraw

You do not have to take part in this research if you do not wish to do so. You may withdraw your participation at any time without any consequence.

Who to contact

If you have any questions, you can ask them now or later.

If you wish to ask questions or make a complaint about the research, you may contact the Lead Researcher: Karsor Kollie (karsorkollie@gmail.com)

If you wish to contact the Institutional Review Board directly, please contact:

Jemee K. Tegli, BBA, Institutional Review Board Coordinator, UL-PIRE AFRICA Center: jktegli@yahoo.com

Thank you for taking time to go over this information sheet. Please feel free to ask further questions you should have any.

Consent Form for CHWs: Focus Group Discussions

CONFIDENTIAL

Activity: Focus group discussion

Title of Project: Identifying the optimal delivery model for the identification, confirmation and referral of NTD cases requiring MMDP services within an integrated health systems approach to NTD care in Liberia

Participant identification number for this study:

If you agree, please put your initials in the box provided as follows:

1. I confirm I have read and understood the information sheet for the above study. I have had the opportunity to consider the information, ask questions and have had these answered satisfactorily. [JV]
2. I understand that participation in this study is voluntary and I am free to withdraw consent at any time, without giving any reason and without any penalties. [JV]
3. I understand that the focus group discussions will be audio-recorded, and notes will be taken. [JV]
4. I understand that data collected during the study, may be looked at by other members of the research team, in Liberia and the UK. I give permission for these individuals to have access to this information. [JV]
5. I understand that recorded data will be collected during the study and deleted from the recorders but stored for 7 years in a secure password protected database that only researchers will have access to. I give permission for these individuals to have access to my records. [JV]
6. I hereby declare that I have not been subjected to any form of coercion in giving this consent. [JV]
7. I agree to take part in this study. [JV]

If no to any of the above the participant cannot take part in the study.

Signing this declaration does not affect your right to decline from taking part in any future study.

Signature of Participant

Signature or Thumbprint	[illegible]	Date of signature	10/10/20

Signature of Witness

Signature or Thumbprint	Kolubah Flomo	Date of signature	Oct. 10, 2020

Signature of Researcher Taking Consent

Signature	[signature]	Date of signature	10/10/20
Name	Colleen Bard Parton		

Jemee K. Tegli, BBA, Institutional Review Board Coordinator, UL-PIRE AFRICA Center: jktegli@yahoo.com

Thank you for taking time to go over this information sheet. Please feel free to ask further questions you should have any.

Consent Form for CHWs: Focus Group Discussions

CONFIDENTIAL

Activity: Focus group discussion

Title of Project: Identifying the optimal delivery model for the identification, confirmation and referral of NTD cases requiring MMDP services within an integrated health systems approach to NTD care in Liberia

Participant identification number for this study:

If you agree, please put your initials in the box provided as follows:

1. I confirm I have read and understood the information sheet for the above study. I have had the opportunity to consider the information, ask questions and have had these answered satisfactorily. [PS]
2. I understand that participation in this study is voluntary and I am free to withdraw consent at any time, without giving any reason and without any penalties. [PS]
3. I understand that the focus group discussions will be audio-recorded, and notes will be taken. [PS]
4. I understand that data collected during the study, may be looked at by other members of the research team, in Liberia and the UK. I give permission for these individuals to have access to this information. [PS]
5. I understand that recorded data will be collected during the study and deleted from the recorders but stored for 7 years in a secure password protected database that only researchers will have access to. I give permission for these individuals to have access to my records. [PS]
6. I hereby declare that I have not been subjected to any form of coercion in giving this consent. [PS]
7. I agree to take part in this study. [PS]

If no to any of the above the participant cannot take part in the study.

Signing this declaration does not affect your right to decline from taking part in any future study.

Signature of Participant

Signature or Thumbprint	[signature]	Date of signature	10/10/20

Signature of Witness

Signature or Thumbprint	Kolubah Flomo	Date of signature	Oct. 10, 2020

Signature of Researcher Taking Consent

Signature	[illegible]	Date of signature	10/10/20
Name	Colleen B McFarter		

Signature or Thumbprint	Kolubah Flomo	Date of signature	Oct. 10, 2020

Signature of Researcher Taking Consent

Signature	[illegible]	Date of signature	10/10/20
Name	Colleen B McPartlin		

Jemee K. Tegli, BBA, Institutional Review Board Coordinator, UL-PIRE AFRICA Center: jktegli@yahoo.com

Thank you for taking time to go over this information sheet. Please feel free to ask further questions you should have any.

Consent Form for CHWs: Focus Group Discussions

CONFIDENTIAL

Activity: Focus group discussion

Title of Project: Identifying the optimal delivery model for the identification, confirmation and referral of NTD cases requiring MMDP services within an integrated health systems approach to NTD care in Liberia

Participant identification number for this study:

If you agree, please put your initials in the box provided as follows:

1. I confirm I have read and understood the information sheet for the above study. I have had the opportunity to consider the information, ask questions and have had these answered satisfactorily. [KM]
2. I understand that participation in this study is voluntary and I am free to withdraw consent at any time, without giving any reason and without any penalties. [KM]
3. I understand that the focus group discussions will be audio-recorded, and notes will be taken. [KM]
4. I understand that data collected during the study, may be looked at by other members of the research team, in Liberia and the UK. I give permission for these individuals to have access to this information. [KM]
5. I understand that recorded data will be collected during the study and deleted from the recorders but stored for 7 years in a secure password protected database that only researchers will have access to. I give permission for these individuals to have access to my records. [KM]
6. I hereby declare that I have not been subjected to any form of coercion in giving this consent. [KM]
7. I agree to take part in this study. [KM]

If no to any of the above the participant cannot take part in the study.

Signing this declaration does not affect your right to decline from taking part in any future study.

Signature of Participant

Signature or<br>Thumbprint	[signature]	Date of signature	10/10/20

Signature of Witness

Jemee K. Tegli, BBA, Institutional Review Board Coordinator, UL-PIRE AFRICA Center: jktegli@yahoo.com

Thank you for taking time to go over this information sheet. Please feel free to ask further questions you should have any.

Consent Form for CHWs: Focus Group Discussions

CONFIDENTIAL

Activity: Focus group discussion

Title of Project: Identifying the optimal delivery model for the identification, confirmation and referral of NTD cases requiring MMDP services within an integrated health systems approach to NTD care in Liberia

Participant identification number for this study:

If you agree, please put your initials in the box provided as follows:

1. I confirm I have read and understood the information sheet for the above study. I have had the opportunity to consider the information, ask questions and have had these answered satisfactorily. [PS]
2. I understand that participation in this study is voluntary and I am free to withdraw consent at any time, without giving any reason and without any penalties. [PS]
3. I understand that the focus group discussions will be audio-recorded, and notes will be taken. [PS]
4. I understand that data collected during the study, may be looked at by other members of the research team, in Liberia and the UK. I give permission for these individuals to have access to this information. [PS]
5. I understand that recorded data will be collected during the study and deleted from the recorders but stored for 7 years in a secure password protected database that only researchers will have access to. I give permission for these individuals to have access to my records. [PS]
6. I hereby declare that I have not been subjected to any form of coercion in giving this consent. [PS]
7. I agree to take part in this study. [PS]

If no to any of the above the participant cannot take part in the study.

Signing this declaration does not affect your right to decline from taking part in any future study.

Signature of Participant

Signature or Thumbprint	[signature]	Date of signature	10/6/20

Signature of Witness

Signature or Thumbprint	Anna Kollie	Date of signature	Oct 6, 2020

Signature of Researcher Taking Consent

Signature	[illegible]	Date of signature	10/6/20
Name	Colleen BAIC Parker		

Signature or<br>Thumbprint	Anna Kollie	Date of signature	Oct 6, 2020

Signature of Researcher Taking Consent

Signature	[illegible]	Date of signature	10/6/20
Name	Colleen B McPartie		

Jemee K. Tegli, BBA, Institutional Review Board Coordinator, UL-PIRE AFRICA Center: jktegli@yahoo.com

Thank you for taking time to go over this information sheet. Please feel free to ask further questions you should have any.

Consent Form for CHWs: Focus Group Discussions

CONFIDENTIAL

Activity: Focus group discussion

Title of Project: Identifying the optimal delivery model for the identification, confirmation and referral of NTD cases requiring MMDP services within an integrated health systems approach to NTD care in Liberia

Participant identification number for this study:

If you agree, please put your initials in the box provided as follows:

1. I confirm I have read and understood the information sheet for the above study. I have had the opportunity to consider the information, ask questions and have had these answered satisfactorily. FD

2. I understand that participation in this study is voluntary and I am free to withdraw consent at any time, without giving any reason and without any penalties. FD

3. I understand that the focus group discussions will be audio-recorded, and notes will be taken. FD

4. I understand that data collected during the study, may be looked at by other members of the research team, in Liberia and the UK. I give permission for these individuals to have access to this information. FD

5. I understand that recorded data will be collected during the study and deleted from the recorders but stored for 7 years in a secure password protected database that only researchers will have access to. I give permission for these individuals to have access to my records. FD

6. I hereby declare that I have not been subjected to any form of coercion in giving this consent. FD

7. I agree to take part in this study. FD

If no to any of the above the participant cannot take part in the study.

Signing this declaration does not affect your right to decline from taking part in any future study.

Signature of Participant

Signature or Thumbprint	[signature]	Date of signature	6/10/20

Signature of Witness

Jemee K. Tegli, BBA, Institutional Review Board Coordinator, UL-PIRE AFRICA Center: jktegli@yahoo.com

Thank you for taking time to go over this information sheet. Please feel free to ask further questions you should have any.

Consent Form for CHWs: Focus Group Discussions

CONFIDENTIAL

Activity: Focus group discussion

Title of Project: Identifying the optimal delivery model for the identification, confirmation and referral of NTD cases requiring MMDP services within an integrated health systems approach to NTD care in Liberia

Participant identification number for this study:

If you agree, please put your initials in the box provided as follows:

1. I confirm I have read and understood the information sheet for the above study. I have had the opportunity to consider the information, ask questions and have had these answered satisfactorily. WB

2. I understand that participation in this study is voluntary and I am free to withdraw consent at any time, without giving any reason and without any penalties. WB

3. I understand that the focus group discussions will be audio-recorded, and notes will be taken. WB

4. I understand that data collected during the study, may be looked at by other members of the research team, in Liberia and the UK. I give permission for these individuals to have access to this information. WB

5. I understand that recorded data will be collected during the study and deleted from the recorders but stored for 7 years in a secure password protected database that only researchers will have access to. I give permission for these individuals to have access to my records. WB

6. I hereby declare that I have not been subjected to any form of coercion in giving this consent. WB

7. I agree to take part in this study. WB

If no to any of the above the participant cannot take part in the study.

Signing this declaration does not affect your right to decline from taking part in any future study.

Signature of Participant

Signature or Thumbprint	[signature]	Date of signature	10/06/20

Signature of Witness

Signature or Thumbprint	Anna Kollie	Date of signature	Oct 6, 2020

Signature of Researcher Taking Consent

Signature	[illegible]	Date of signature	10/16/20
Name	Colleen BAYC Partner		

Jemee K. Tegli, BBA, Institutional Review Board Coordinator, UL-PIRE AFRICA Center: jktegli@yahoo.com

Thank you for taking time to go over this information sheet. Please feel free to ask further questions you should have any.

Consent Form for CHWs: Focus Group Discussions

CONFIDENTIAL

Activity: Focus group discussion

Title of Project: Identifying the optimal delivery model for the identification, confirmation and referral of NTD cases requiring MMDP services within an integrated health systems approach to NTD care in Liberia

Participant identification number for this study:

If you agree, please put your initials in the box provided as follows:

1. I confirm I have read and understood the information sheet for the above study. I have had the opportunity to consider the information, ask questions and have had these answered satisfactorily. [JK]

2. I understand that participation in this study is voluntary and I am free to withdraw consent at any time, without giving any reason and without any penalties. [JK]

3. I understand that the focus group discussions will be audio-recorded, and notes will be taken. [JK]

4. I understand that data collected during the study, may be looked at by other members of the research team, in Liberia and the UK. I give permission for these individuals to have access to this information. [JK]

5. I understand that recorded data will be collected during the study and deleted from the recorders but stored for 7 years in a secure password protected database that only researchers will have access to. I give permission for these individuals to have access to my records. [JK]

6. I hereby declare that I have not been subjected to any form of coercion in giving this consent. [JK]

7. I agree to take part in this study. [JK]

If no to any of the above the participant cannot take part in the study.

Signing this declaration does not affect your right to decline from taking part in any future study.

Signature of Participant

Signature or<br>Thumbprint	James Kai	Date of signature	[signature] 6/10/20

Signature of Witness

Signature or Thumbprint	Anna Kollie	Date of signature	Oct 6, 2020

Signature of Researcher Taking Consent

Signature	[illegible]	Date of signature	10/16/20
Name	Colleen BMcPartlan		

Jemee K. Tegli, BBA, Institutional Review Board Coordinator, UL-PIRE AFRICA Center: jktegli@yahoo.com

Thank you for taking time to go over this information sheet. Please feel free to ask further questions you should have any.

Consent Form for CHWs: Focus Group Discussions

CONFIDENTIAL

Activity: Focus group discussion

Title of Project: Identifying the optimal delivery model for the identification, confirmation and referral of NTD cases requiring MMDP services within an integrated health systems approach to NTD care in Liberia

Participant identification number for this study:

If you agree, please put your initials in the box provided as follows:

1. I confirm I have read and understood the information sheet for the above study. I have had the opportunity to consider the information, ask questions and have had these answered satisfactorily. [CB]
2. I understand that participation in this study is voluntary and I am free to withdraw consent at any time, without giving any reason and without any penalties. [CB]
3. I understand that the focus group discussions will be audio-recorded, and notes will be taken. [CB]
4. I understand that data collected during the study, may be looked at by other members of the research team, in Liberia and the UK. I give permission for these individuals to have access to this information. [CB]
5. I understand that recorded data will be collected during the study and deleted from the recorders but stored for 7 years in a secure password protected database that only researchers will have access to. I give permission for these individuals to have access to my records. [CB]
6. I hereby declare that I have not been subjected to any form of coercion in giving this consent. [CB]
7. I agree to take part in this study. [CB]

If no to any of the above the participant cannot take part in the study.

Signing this declaration does not affect your right to decline from taking part in any future study.

Signature of Participant

Signature or Thumbprint	[signature]	Date of signature	10/6/20

Signature of Witness

Signature or<br>Thumbprint	Anna Kollie	Date of signature	Oct 4, 2020

Signature of Researcher Taking Consent

Signature	[illegible]	Date of signature	10/16/20
Name	Colleen B McPar[illegible]		

Jemee K. Tegli, BBA, Institutional Review Board Coordinator, UL-PIRE AFRICA Center: jktegli@yahoo.com

Thank you for taking time to go over this information sheet. Please feel free to ask further questions you should have any.

Consent Form for CHWs: Focus Group Discussions

CONFIDENTIAL

Activity: Focus group discussion

Title of Project: Identifying the optimal delivery model for the identification, confirmation and referral of NTD cases requiring MMDP services within an integrated health systems approach to NTD care in Liberia

Participant identification number for this study:

If you agree, please put your initials in the box provided as follows:

1. I confirm I have read and understood the information sheet for the above study. I have had the opportunity to consider the information, ask questions and have had these answered satisfactorily. [P.K]

2. I understand that participation in this study is voluntary and I am free to withdraw consent at any time, without giving any reason and without any penalties. [P.K]

3. I understand that the focus group discussions will be audio-recorded, and notes will be taken. [P.K]

4. I understand that data collected during the study, may be looked at by other members of the research team, in Liberia and the UK. I give permission for these individuals to have access to this information. [P.K]

5. I understand that recorded data will be collected during the study and deleted from the recorders but stored for 7 years in a secure password protected database that only researchers will have access to. I give permission for these individuals to have access to my records. [P.K]

6. I hereby declare that I have not been subjected to any form of coercion in giving this consent. [P.K]

7. I agree to take part in this study. [P.K]

If no to any of the above the participant cannot take part in the study.

Signing this declaration does not affect your right to decline from taking part in any future study.

Signature of Participant

Signature or Thumbprint	P. King	Date of signature	Oct 8, 2020

Signature of Witness

Signature or<br>Thumbprint	JC Reed.	Date of signature<br>Oct 8, 2020	

Signature of Researcher Taking Consent

Signature	JGobeh	Date of signature<br>Oct. 8, 2020	
Name	Jallah Z. Gobeh		

Jemee K. Tegli, BBA, Institutional Review Board Coordinator, UL-PIRE AFRICA Center: jktegli@yahoo.com

Thank you for taking time to go over this information sheet. Please feel free to ask further questions you should have any.

Consent Form for CHWs: Focus Group Discussions

CONFIDENTIAL

Activity: Focus group discussion

Title of Project: Identifying the optimal delivery model for the identification, confirmation and referral of NTD cases requiring MMDP services within an integrated health systems approach to NTD care in Liberia

Participant identification number for this study:

If you agree, please put your initials in the box provided as follows:

1. I confirm I have read and understood the information sheet for the above study. I have had the opportunity to consider the information, ask questions and have had these answered satisfactorily. [B.B.W]

2. I understand that participation in this study is voluntary and I am free to withdraw consent at any time, without giving any reason and without any penalties. [BBW]

3. I understand that the focus group discussions will be audio-recorded, and notes will be taken. [B.B.W]

4. I understand that data collected during the study, may be looked at by other members of the research team, in Liberia and the UK. I give permission for these individuals to have access to this information. [B.B.W]

5. I understand that recorded data will be collected during the study and deleted from the recorders but stored for 7 years in a secure password protected database that only researchers will have access to. I give permission for these individuals to have access to my records. [B.B.W]

6. I hereby declare that I have not been subjected to any form of coercion in giving this consent. [BBW]

7. I agree to take part in this study. [B.BW]

If no to any of the above the participant cannot take part in the study.

Signing this declaration does not affect your right to decline from taking part in any future study.

Signature of Participant

Signature or Thumbprint	[signature]	Date of signature	Oct 8, 2020

Signature of Witness

Signature or Thumbprint	ICReed	Date of signature Oct 8, 2020	

Signature of Researcher Taking Consent

Signature	JGobeh	Date of signature	
Name	Jallah Z. Gobeh	Oct. 8, 2020	

Jemee K. Tegli, BBA, Institutional Review Board Coordinator, UL-PIRE AFRICA Center: jktegli@yahoo.com

Thank you for taking time to go over this information sheet. Please feel free to ask further questions you should have any.

Consent Form for CHWs: Focus Group Discussions

CONFIDENTIAL

Activity: Focus group discussion

Title of Project: Identifying the optimal delivery model for the identification, confirmation and referral of NTD cases requiring MMDP services within an integrated health systems approach to NTD care in Liberia

Participant identification number for this study:

If you agree, please put your initials in the box provided as follows:

1. I confirm I have read and understood the information sheet for the above study. I have had the opportunity to consider the information, ask questions and have had these answered satisfactorily. [R.O.M]
2. I understand that participation in this study is voluntary and I am free to withdraw consent at any time, without giving any reason and without any penalties. [R.O.M]
3. I understand that the focus group discussions will be audio-recorded, and notes will be taken. [R.O.M]
4. I understand that data collected during the study, may be looked at by other members of the research team, in Liberia and the UK. I give permission for these individuals to have access to this information. [R.O.M]
5. I understand that recorded data will be collected during the study and deleted from the recorders but stored for 7 years in a secure password protected database that only researchers will have access to. I give permission for these individuals to have access to my records. [R.O.M]
6. I hereby declare that I have not been subjected to any form of coercion in giving this consent. [R.O.M]
7. I agree to take part in this study. [R.O.M]

If no to any of the above the participant cannot take part in the study.

Signing this declaration does not affect your right to decline from taking part in any future study.

Signature of Participant

Signature or Thumbprint	R.O.M	Date of signature	Oct. 8, 2020

Signature of Witness

Signature or<br><br>Thumbprint	IC Reed	Date of signature<br>Oct 8, 2020	

Signature of Researcher Taking Consent

Signature	[illegible]	Date of signature<br>Oct. 8, 2020	
Name	Jallah Z. Gobeh		

Jemee K. Tegli, BBA, Institutional Review Board Coordinator, UL-PIRE AFRICA Center: jktegli@yahoo.com

Thank you for taking time to go over this information sheet. Please feel free to ask further questions you should have any.

Consent Form for CHWs: Focus Group Discussions

CONFIDENTIAL

Activity: Focus group discussion

Title of Project: Identifying the optimal delivery model for the identification, confirmation and referral of NTD cases requiring MMDP services within an integrated health systems approach to NTD care in Liberia

Participant identification number for this study:

If you agree, please put your initials in the box provided as follows:

1. I confirm I have read and understood the information sheet for the above study. I have had the opportunity to consider the information, ask questions and have had these answered satisfactorily. [SSW]

2. I understand that participation in this study is voluntary and I am free to withdraw consent at any time, without giving any reason and without any penalties. [SSW]

3. I understand that the focus group discussions will be audio-recorded, and notes will be taken. [SSW]

4. I understand that data collected during the study, may be looked at by other members of the research team, in Liberia and the UK. I give permission for these individuals to have access to this information. [SSW]

5. I understand that recorded data will be collected during the study and deleted from the recorders but stored for 7 years in a secure password protected database that only researchers will have access to. I give permission for these individuals to have access to my records. [SSW]

6. I hereby declare that I have not been subjected to any form of coercion in giving this consent. [SSW]

7. I agree to take part in this study. [S.SW]

If no to any of the above the participant cannot take part in the study.

Signing this declaration does not affect your right to decline from taking part in any future study.

Signature of Participant

Signature or Thumbprint	S.S.W	Date of signature	Oct 8, 2020

Signature of Witness

Signature or<br>Thumbprint	ICReed	Date of signature<br>Oct 8, 2020	

Signature of Researcher Taking Consent

Signature	JZGobeh	Date of signature<br>Oct. 8, 2020	
Name	Jallah Z. Gobeh		

Jemee K. Tegli, BBA, Institutional Review Board Coordinator, UL-PIRE AFRICA Center: jktegli@yahoo.com

Thank you for taking time to go over this information sheet. Please feel free to ask further questions you should have any.

Consent Form for CHWs: Focus Group Discussions

CONFIDENTIAL

Activity: Focus group discussion

Title of Project: Identifying the optimal delivery model for the identification, confirmation and referral of NTD cases requiring MMDP services within an integrated health systems approach to NTD care in Liberia

Participant identification number for this study:

If you agree, please put your initials in the box provided as follows:

1. I confirm I have read and understood the information sheet for the above study. I have had the opportunity to consider the information, ask questions and have had these answered satisfactorily. [K.M]

2. I understand that participation in this study is voluntary and I am free to withdraw consent at any time, without giving any reason and without any penalties. [K.M]

3. I understand that the focus group discussions will be audio-recorded, and notes will be taken. [K.M]

4. I understand that data collected during the study, may be looked at by other members of the research team, in Liberia and the UK. I give permission for these individuals to have access to this information. [K.M]

5. I understand that recorded data will be collected during the study and deleted from the recorders but stored for 7 years in a secure password protected database that only researchers will have access to. I give permission for these individuals to have access to my records. [K.M]

6. I hereby declare that I have not been subjected to any form of coercion in giving this consent. [K.M]

7. I agree to take part in this study. [K M]

If no to any of the above the participant cannot take part in the study.

Signing this declaration does not affect your right to decline from taking part in any future study.

Signature of Participant

Signature or Thumbprint	K.M	Date of signature	Oct. 8, 2020

Signature of Witness

Signature or<br>Thumbprint	ICRead .	Date of signature<br>Oct 8, 2020	

Signature of Researcher Taking Consent

Signature	JGobeh	Date of signature<br>oct. 8, 2020	
Name	Jallah Z. Gobeh		

Jemee K. Tegli, BBA, Institutional Review Board Coordinator, UL-PIRE AFRICA Center: jktegli@yahoo.com

Thank you for taking time to go over this information sheet. Please feel free to ask further questions you should have any.

Consent Form for CHWs: Focus Group Discussions

CONFIDENTIAL

Activity: Focus group discussion

Title of Project: Identifying the optimal delivery model for the identification, confirmation and referral of NTD cases requiring MMDP services within an integrated health systems approach to NTD care in Liberia

Participant identification number for this study:

If you agree, please put your initials in the box provided as follows:

1. I confirm I have read and understood the information sheet for the above study. I have had the opportunity to consider the information, ask questions and have had these answered satisfactorily. [OB]
2. I understand that participation in this study is voluntary and I am free to withdraw consent at any time, without giving any reason and without any penalties. [OB]
3. I understand that the focus group discussions will be audio-recorded, and notes will be taken. [OB]
4. I understand that data collected during the study, may be looked at by other members of the research team, in Liberia and the UK. I give permission for these individuals to have access to this information. [OB]
5. I understand that recorded data will be collected during the study and deleted from the recorders but stored for 7 years in a secure password protected database that only researchers will have access to. I give permission for these individuals to have access to my records. [OB]
6. I hereby declare that I have not been subjected to any form of coercion in giving this consent. [OB]
7. I agree to take part in this study. [OB]

If no to any of the above the participant cannot take part in the study.

Signing this declaration does not affect your right to decline from taking part in any future study.

Signature of Participant

Signature or Thumbprint		Date of signature	10/10/20

Signature of Witness

Signature or Thumbprint	Kolubah Flomo	Date of signature	Oct. 10, 2020

Signature of Researcher Taking Consent

Signature	[illegible]	Date of signature	10/10/20
Name	Colleen B McPartlan		

Jemee K. Tegli, BBA, Institutional Review Board Coordinator, UL-PIRE AFRICA Center: jktegli@yahoo.com

Thank you for taking time to go over this information sheet. Please feel free to ask further questions you should have any.

Consent Form for CHWs: Focus Group Discussions

CONFIDENTIAL

Activity: Focus group discussion

Title of Project: Identifying the optimal delivery model for the identification, confirmation and referral of NTD cases requiring MMDP services within an integrated health systems approach to NTD care in Liberia

Participant identification number for this study:

If you agree, please put your initials in the box provided as follows:

1. I confirm I have read and understood the information sheet for the above study. I have had the opportunity to consider the information, ask questions and have had these answered satisfactorily. [BT]
2. I understand that participation in this study is voluntary and I am free to withdraw consent at any time, without giving any reason and without any penalties. [BT]
3. I understand that the focus group discussions will be audio-recorded, and notes will be taken. [BT]
4. I understand that data collected during the study, may be looked at by other members of the research team, in Liberia and the UK. I give permission for these individuals to have access to this information. [BT]
5. I understand that recorded data will be collected during the study and deleted from the recorders but stored for 7 years in a secure password protected database that only researchers will have access to. I give permission for these individuals to have access to my records. [BT]
6. I hereby declare that I have not been subjected to any form of coercion in giving this consent. [BT]
7. I agree to take part in this study. [BT]

If no to any of the above the participant cannot take part in the study.

Signing this declaration does not affect your right to decline from taking part in any future study.

Signature of Participant

Signature or Thumbprint	B. Tbm	Date of signature	10/10/20

Signature of Witness

Signature or<br>Thumbprint	Kolubah Flomo	Date of signature	Oct. 10, 2020

Signature of Researcher Taking Consent

Signature	[illegible]	Date of signature	10/10/20
Name	Colleen [illegible] Parker		
