# Supplementary material for: The development, implementation, and evaluation of an optimal model for the case detection, referral, and case management of Neglected Tropical Diseases
Source: PLoS One. 2023 May 10;18(5):e0283856. doi: 10.1371/journal.pone.0283856 (PMC10171595; doi:10.1371/journal.pone.0283856)
Supplement: S5 File — (PDF) [file pone.0283856.s005.pdf]

Theme	Illustrative quotes
Inclusion and collaboration	*'But what was interesting at the end…the formative phase, actually gave birth to the implementation phase. Because we had feedback from the formative phase, and that feedback took into consideration the patients, we brought in some of the patients, to lead us into the intervention phase. And we brought in the community health.' (NTD Programme, Reflexive Session)*<br><br>*'Considering holding the meeting outside of Monrovia…The meeting being held in Monrovia meant that the number of affected persons and community volunteers that could participate was sometimes limited.' (Researcher, Reflexive Session)*
Training	*'The material was very helpful...because you can go talk to someone for example you ask a person with Buruli ulcer. Then they start to scratch their head what is Buruli ulcer. But if they see the photo this is how Buruli ulcer looks like, this is hydrocele. Because in our scene they say seeing is believing. So when they see the photo and the trainer they did their best by teaching those important tools make our work easy.' (CHW, FGD, Bong)*<br><br>*'The training did something good for me because those things we use to see them we will go to herbalist and to do country medicine. But during the training we were told if we find case like this you refer them. And it helps me to also identify NTDs.' (CHW, FGD, Bong)*<br><br>*'I got to know the importance of NTDs. For the past we used to see these cases but it was not to our knowledge that it is a problem that can be treated. Sometimes we shift the blame on witchcraft' (CHW, FGD, Bong)*<br><br>*'What I will like to add to this training is it should not be only one day, it should take two to three days that we will be able to go through the modules lengthily that we understand it best (CHW, FGD, Bong)*
Trust and Rapport with Communities: The essential role of peer advocates	*'The thing I like about it is that it gives you more respect that you learned something and when you talk to somebody that person will listen to you. (Reflexive observation, Peer advocate training)*<br>*'The impression of the PAs while conducting active case search was very impressive. They are eager and committed to work with CHAs/CHVs within their assigned communities and to help as many persons as possible by identifying cases and referring them to the health facilities. (Reflexive observation, Peer advocate training)*<br><br>*'It's a good thing because any condition you facing when you are training to know about that condition it will help you. you will be able to help yourself to handle it you will also be able to bring people up to talk to people that get condition and compare your case with them. and say you see myself I had this problem but this is the action I took and this is where I am today so I will encourage you. (CHSS, KII, Bong).*

Gender Balance	*'There can be challenges because sometimes the male will not want to explain their problems to the female, they will not want to disclose to you. (CHW, FGD, Bong)*<br><br>*'The different way there is for me in my community, when I went places, first of all I will go to the husband or boyfriend and say, friend, I am suspecting certain thing on your wife which we are looking for. Would you allow me sit with her and talk or you both are there so we can talk? For a man he is a man like me so I will say, my man, come and let's lecture and he will do that, so it just man and not in a harsh manner but in a polite way. But for female you link that with the boyfriend before you can start. (CHW, FGD, Bong)*<br><br>*'Like my colleague said, when it comes to the gender issue getting somebody home the person who has this sickness getting in their home we should get there with respect. Number one thing that trust we the CHV trust they should trust us. Have that strong relationship with the people and also trust to the matter for them. (CHW, FGD, Bong)*
Supervision	*'I feel happy because they are directing me and telling me what to do. If I have done wrong they will tell me, yes, this is wrong; they are putting me on my guard. I feel well as they are giving me some verbal thoughts in the regular way to enable do the work good' (Peer advocate, FDG, Bong)*<br><br>*'When it comes to supervision in the facility, we monitor our CHV on a daily basis. Because we communicate with them, we move with them face to face. There are some cases that they will call you to go and see. So we supervise them by going to them, by communicating with them on cellphone and so.' (CHSS, KII, Bong).*
Referral	*' [The feedback form]…has been very effective for those that have been looking for cases. At time we get challenges, because at time the facility will run out of form but still it has been effective.' (CHSS, KII, Bong).*<br><br>*'When we refer them for treatment at the clinic. To come sometime it can be very hard because they can say we not get money. When we go to the clinic the people will say we must buy drugs. So why…there is no need to go there. It's better for me to go to another drug store but still we have to encourage them. And they can come.' (CHW, FGD, Bong).*
Remuneration	*'Before they used to tell the people when they find the case they were going to give them 5 dollars. So, the people used to find the case when they bring it then they can motivate them with that 5 dollars. ' (CHW, FGD, Bong).*<br><br>*'…sometime when you bring more cases they are motivated with five dollars. But at least if they can put the motivation up that it will help them.' (CHW, FGD, Bong).*<br><br>*'Another is this five dollar issue. The five dollar should come on time.' (CHSS, KII, Bong).*
