# Supplementary material for: The development, implementation, and evaluation of an optimal model for the case detection, referral, and case management of Neglected Tropical Diseases
Source: PLoS One. 2023 May 10;18(5):e0283856. doi: 10.1371/journal.pone.0283856 (PMC10171595; doi:10.1371/journal.pone.0283856)
Supplement: S1 Appendix — (PDF) [file pone.0283856.s006.pdf]

Supervision Tools

NTDs Supervision Tool – Regional Supervisors

Month Day Year

Supervisee's:
Name Sex M F Position

Supervisor's Name Sex M F Position

Supervision: Start Time End Time Venue

#	QUESTION/QUERRY	FINDING	ACTION TAKEN/ ACTION POINT
1.1	Regional supervisor has completed report of visit at each level during supervision<br>*Review completed reports for each visit.*		
1.2	Regional supervisor has identified facilities with issues as per the data and listed those to be visited during supervision<br>*Review facility listing and confirm issues identified by Regional supervisor.*		
2.1	Regional supervisor has submitted non-DHIS2 reports from NTD FPs on time.<br>*Ensure the reports are completed*		
2.2	Regional supervisor has the updated report from the laboratory on the number of cases confirmed out of those sampled.<br>*Confirm with report from the laboratory*		
3.1	Regional supervisor has an updated list of health facilities with stock out and the specific supplies needed.		
3.2	Number of health facilities with zero report for any of the disease conditions over the last quarter visited by the regional supervisor<br>*Confirm with supervision report*		
4.1	Regional supervisor has identified issues with CHSS report and that of the CHA/CHVs and have taken actions to correct them		
4.2	Regional supervisor has reviewed all reports from NTD FPs and have confirm for completeness and accuracy		
4.3	Number of NTD FP reports that matches the DHIS2 reports from the facilities.		
4.4	Regional supervisor regularly checks in with the NTD focal persons to identify and address any issues		

NTDs Supervision Tool – OIC/2nd Screener

County H. District Health facility

Month Day Year OIC/2nd Screener ID

Supervisee's:
Name Sex M F Position

Supervisor's Name Sex M F Position

Supervision: Start Time End Time Venue

#	QUESTION/QUERRY	FINDING	ACTION TAKEN/ ACTION POINT
1.1	Number of cases clinically diagnosed per disease condition in the last month. *Review health facility ledger and patient card to confirm each case reported*		
1.2	Number of BU samples collected and sent to the National reference laboratory for confirmation. *Review all laboratory forms for confirmation*		
2.1	Number of BU samples confirmed by PCR		
2.2	For cases confirmed by the laboratory, was there any feedback to the CHSS/CHC for follow up at the community level? *Probe for evidence of feedback to the community*		
3.1	Number of cases confirmed who have completed treatment on time in the last month. *Review health facility ledger to ensure all patients diagnosed have complete data*		
3.2	Number of cases currently on treatment		
4.1	Were there health talks conducted in the last month that included CM? *Probe for the number of health talks and number of participants. Confirm by reviewing the health talk schedule in the facility*		
4.2	Are there CM-NTDs posters in the facility? If so, where are they located? *Ensure the posters are present in the health facility*		

4.3	Are there CM-NTDs posters in the community? If so, where are they located? *Go into the community to confirm the presence of the posters.*		
4.4	Does the facility currently have stock of essential drugs for treatment of CM-NTDs cases? *Probe to see the current stock of drugs.*		
4.5	Does the facility have essential supplies including: dressing materials and home-based self-care kit? *Probe to see the current stock*		
4.6	Is the OIC/2nd screener trained on the management of CM-NTDs? *Probe to understand how clinically diagnosis is done for each patient.*		
5.1	Are CM-NTDs patients being managed within the general rooms or separately? *This is important to understand any discrimination at the facility level.*		
5.2	Does the facility receive feedback on time from the laboratory? *Probe to confirm how long it takes to from the time samples are sent to when the feedback is sent.*		

NTDs Supervision Tool – DSO

County H. District

Month Day Year

Supervisee's:
Name Sex M F Position

Supervisor's Name Sex M F Position

Start time

#	QUESTION/QUERRY	FINDING	ACTION TAKEN/ ACTION POINT
1.1	Number of health facilities visited in the last month *Review reports from health facility and confirm with health facility register during supervision at the facility.*		
1.2	Number of health facilities with completed and timely reports. *Review reports from health facilities and confirm*		
2.1	Number of samples collected for BU and yaws during the last month. *Review laboratory form to confirm*		
2.2	Are there any challenges or constraints with the collection and transportation of samples?		
3.1	Number of health facilities with last month report in the DHIS2. *Probe to see the DHIS2 platform to confirm*		

3.2	During the last supervision was there any issue around diagnosis and management of NTDs that was observed?		

NTDs Supervision Tool – NTD FP

County

Month Day Year

Supervisee's:
Name Sex M F Position

Supervisor's Name Sex M F Position

Supervision: Start Time End Time Venue

#	QUESTION/QUERRY	FINDING	ACTION TAKEN/ ACTION POINT
1.1	How many facilities have been visited in the last month? *Probe to review supervision report for each facility visited to confirm.*		
1.2	Was supervision conducted with the community health focal person? If not, why?		
2.1	Number of health facilities with completed reports? *Review report from the districts to confirm*		
2.2	Number of suspected cases referred who visited the health facility? *Review CHSS report to confirm*		
3.1	Number of suspected cases counselled by CHSS who refused to visit the health facility. *Review CHSS report to confirm*		
3.2	Number of patients diagnosed who were not referred by CHA/CHVs that are being followed at the community level.		
4.1	Number of patients linked to peer advocates.		
4.2	Number of CM-NTDs cases clinically diagnosed in the last month. *Please list per disease condition*		
4.3	Number of BU samples collected?		
4.4	Number of BU samples confirmed by PCR. *Confirm with laboratory form*		
4.5	Number of yaws samples collected		
4.6	Number of yaws samples confirmed		
5.1	Number of leprosy patients whose contacts have been traced. *Confirm with NTDs contact tracing forms*		
5.2	Number of yaws patients whose contacts have been traced. *Confirm with NTDs contact tracing forms*		
5.3	Number of patients who have completed treatment per disease condition in the last month.		

5.4	Number of patients currently on treatment per disease condition. *Confirm with health facility ledger and health facility reports*		
5.5	Number of defaulters. *Confirm with defaulters contact tracing form*		
5.6	Number of health facilities with stock of essential drugs and supplies for NTDs. *Confirm by visiting selected facilities and seeing current stock of CM drugs and management materials*		
5.7	Number of health facilities with needed CM tools such as: integrated health facility ledger, laboratory forms, community feedback forms, BU scoring form for diagnosis etc. *Confirm by visiting selected facilities and seeing current stock of CM tool*.		
5.8	Number of health facilities coached/mentored by NTD FP in the last month. *Confirm by reviewing the supervision reports from those facilities*		
5.9	Number of communities visited and reports verified by the CHSS during the last month? *Confirm by seeing supervision reports*		
6.0	How many of the CHSS reports did not match the CHA/CHV reports? *Confirm by seeing the CHSS and CHA/CHV reports that conflict with each other.*		
6.1	Number of counties monthly meeting minutes with NTDs		
6.2	Number of CHA/CHVs who confirm receiving the $5 incentive for confirmed cases. *Review feedback forms*		
6.3	Number of health facilities whose ledger have complete data for all NTD patients.		
6.4	Number of health facilities with peer advocates established and active. *Confirm by meeting representatives of the peer advocate group.*		
6.5	Any other issues around discrimination at the health facility for example: collecting more money from NTDs patients for dressing, managing NTD patients in separate room from the general ward etc.		
6.6	Number of health facilities with last month report in the DHIS2. *Confirm at the district level*		

M

☐

Community Health Services Division
Ministry of Health
Monrovia, Liberia
CHA/CHV Supervision Tool

Date: ___________________ Time Supervision
Started: _______ Time Session Ended:______________
Supervisor Name ____________________ Title____________________
County: __________________ District: _________________________
Name of CHV:_________________________
Name of Health Facility_______________________
Community:____________________

When doing monthly supervision, it is important that supervisors review what is being done in the community by the health assistant/volunteer and also provide teaching and reinforcement. Please have the CHAs/CHVs bring their ledgers and all recording reporting forms in their backpacks to conduct a private teaching session with them to review their documents. If the community has more than one CHV, parts of this review can be done together to assist and support them in their learning. The meeting with a member of the CHC should be conducted when possible with CHAs /CHVs present. After these meetings, conduct an observation of the community and please take time to do a random visit of at least 6 households. Community information forms only need to be completed by one CHA/CHV and stated as such on the other forms.
Ledger review: Review all CHAs/CHV's community ledgers.

Indicator **Means of Verification**

Referral and Feedback **# of cases**

1.1 Number of Leprosy cases referred?	
1.2 Number of Yaws cases identified referred?	
1.3 Number of BU cases referred?	
1.4 Number of elephantiasis cases referred?	
1.5 Number of Hydrocele cases?	
**2.0 Were there cases referred who did not visit the health facility during last month?**	
**2.1 How many did not reach the facility?**	
**2.2 Ask the CHA/CHV what were the reasons why some suspects did not reach the health facility and write it down**	
**2.3 Ask the CHA/CHV what actions they took to get the suspect to the health facility**	
**3.0 Number of counter referral forms received by CHA/CHVs for each disease condition**	
3. 1 Leprosy?	
3.2 Yaws?	
3.3 BU?	
3.4 Elephantiasis	
2.5 Hydrocele?	
**Number of new patients who were visited in the last month**	
**3.0 Number of patients on treatment visited in the last month**	
3.1 Number of Leprosy patients visited in the last month?	
3.2 Number of yaws patients visited in the last month?	
3.3 Number of BU patients visited at least twice in the last month?	
3.4 Number of Elephantiasis patients visited in the last month?	
3, 5 Number of Hydrocele patients visited in the last month?	

**How many elephantiasis patients were practicing home based self-care in the last month?**	
**Number of leprosy patients whose contacts were traced in the last month**	
**Number of Yaws patients whose contacts were traced in the last month**	
**Number of defaulters traced in the last month**	
**4.0 Ask the CHA/**CHV **for the availability of the following CBIS forms and circle if it is available**	**Circle the letter that is available**
a, Routine Visit, b, Community Trigger, c, Referral form, d, Case Management Ledger, f, monthly Service report,	
Are there any other issues?	

CHSD/NTDs Supervision Tool – CHSS/CHC

County H. District Community

Month Day Year CHSS/CHC ID SUPVR. ID

Supervisee's:
Name Sex M F Position

Supervisor's Name Sex M F Position

Supervision: Start Time End Time Venue

#	QUESTION/QUERRY	FINDING	ACTION TAKEN/ ACTION POINT
1.1	CHSS/CHC has record of suspected CM NTDs cases who did not visit the HF within the last month<br>*Probe for # of suspects on record. Check CHA/CHV reports to confirm cases who didn't visit the facility then review CHSS ledger for summary of visit. Probe for reasons why they did not visit.*		
1.2	CHSS/CHC has record of all CM cases referred who visited the health facility during the last month. (*Verify the listing)*		
	CHSS/CHC has record of all suspected cases diagnosed within the last month<br>(*Verify the record)*		
	CHSS/CHC has record of CM cases diagnosed that were not referred by CHA/CHV but are being followed up by the CHA/CHV		
	CHSS/CHC has record of suspected and confirmed cases who have been linked to the peer advocates within the last month		
	Number of patients on treatment during the last month		

	(*Verify using health facility ledger)*		
	Number of Elephantiasis patients practicing self-care in the last month. (*Review CHA/CHV reports to confirm)*		
2.1	CHSS/CHC has record of all CM patients who completed during the last month..<br>*Probe for # of patients who have completed treatment per disease condition).*		
2.2	CHSS/CHC has followed-up on all patients who were on treatment during the last month.<br>*Probe for # of patients followed-up.*		
3.1	CHSS/CHC has identified patients who defaulted and were lost to follow-up during the last month.<br>*Probe for # of patients*.		
3.2	Number of defaulters and lost to follow-up who have been traced.<br>*Probe for # traced. (Review contact tracing form to confirm)*		
4.1	CHSS/CHC has record of all Leprosy and Yaws patients diagnosed during the last month.<br>*Probe for number of Leprosy and Yaws Patients*.		
4.2	CHSS/CHC has records of the number of contacts of Leprosy and Yaws patients during the last month.<br>*Probe for number of contacts generated. Review NTDs contact tracing form to confirm number of contacts for each patient*		
4.3	CHSS/CHC has ensured that contact tracing has been conducted for all Leprosy and Yaws contacts generated during the last month.<br>*Probe for number of contacts traced.*		
4.4	CHSS/CHC has record of all CHAs/CHVs within his/her catchment area.		
4.5	CHSS/CHC conducted on-site coaching/mentoring for CHAs/CHVs during the last month.<br>*Review all supervision forms for each CHA/CHV within the catchment area*		
4.6	List names of CHAs/CHVs coached/mentored.		
5.1	Conduct CHA/CHV Spot Check:<br>*Ask CHSS/CHC to invite at least one CHA and one CHV to speak to you in the absence of the CHSS/CHC. Then inquire from the CHA/CHV any issues that need to be addressed including: transportation for confirmed cases, link to peer advocate and patients willingness, and job aids etc.*		
5.3	Number of counter referral forms received by CHA/CHV in the last month		
5.4	CHSS has record of new patients visited in the last month		
5.5	CHSS has record that all CHA/CHV have available CBIS forms		
