# Supplementary material for: The development, implementation, and evaluation of an optimal model for the case detection, referral, and case management of Neglected Tropical Diseases
Source: PLoS One. 2023 May 10;18(5):e0283856. doi: 10.1371/journal.pone.0283856 (PMC10171595; doi:10.1371/journal.pone.0283856)
Supplement: S2 Appendix — (PDF) [file pone.0283856.s007.pdf]

CERTIFICATE OF TRAINING

This is to certify that

. . . .

Successfully completed the training course **'Module 4: Special Programs Integrated Case Management'**

Awarded:

Karsor Kollie
Director of the Neglected Tropical Disease Program

Emerson Rogers
Case Management Coordinator
