# Supplementary material for: The development, implementation, and evaluation of an optimal model for the case detection, referral, and case management of Neglected Tropical Diseases
Source: PLoS One. 2023 May 10;18(5):e0283856. doi: 10.1371/journal.pone.0283856 (PMC10171595; doi:10.1371/journal.pone.0283856)
Supplement: S3 Appendix — (PDF) [file pone.0283856.s008.pdf]

MODULE 4: SPECIAL PROGRAMS

INTEGRATED CASE MANAGEMENT (CM): LEPROSY, BURULI ULCER, ELEPHANTIASIS, HYDROCELE AND YAWS

COMMUNITY HEALTH ASSISTANT AND VOLUNTEER HANDBOOK

MINISTRY OF HEALTH

REPUBLIC OF LIBERIA

COMMUNITY HEALTH DIVISION, NEGLECTED TROPICAL DISEASES

& TRAINING DIVISIONS

MINISTY OF HEALTH, REPUBLIC OF LIBERIA

31 OCTOBER 2019

TABLE OF CONTENTS

SESSION	TITLE
Lesson 4.3.1	Pre-test and Overview (30 minutes)
Lesson 4.3.2	Introduction to Integrated Case Management (7 minutes)
Lesson 4.3.3	Leprosy, BU, Elephantiasis, Hydrocele and Yaws Identification, Referral and Management (xx minutes)
Lesson 4.3.4	Practice Session: Leprosy, BU, Elephantiasis, Hydrocele and Yaws Identification, Referral and Management (xx minutes)
Lesson 4.3.5	Supporting People Living with Leprosy, BU, EL, Hydrocele and Yaws and Messages to Reduce Stigma (xx minutes)
Lesson 4.3.6	Essential CHA Module 4 Forms: Review and Practice (xx minutes)
Lesson 4.3.7	Review and Post-Test (xx minutes)

REFERENCES	ACCRONYMS & ABBREVIATIONS
• http://www.aho.afro.who.int/profiles_information/index.php/Liberia: Analytical_summary_-_Neglected_tropical_diseases<br>• MOH (2019). Essential Package of Health Care Services. Retrieved from http://www.moh.gov.lr/doc/Final%20EPHS.pdf.<br>• Basic Counseling Skills: MOH Liberia, Leprosy Prevalence and Incidence 2009 http://www.buildingpeace.org/sites/default/files	• **NTD-** Neglected Tropical Diseases<br>• **CM-** Integrated Case Management<br>• **LE-**Leprosy<br>• **BU-**Buruli Ulcer

/Core%20Principles%20of%20Active%20Listening %20Handout.pdf	• **EL-**Elephantiasis<br>• **H-**Hydrocele<br>• **YA-**Yaws

MODULE 4: SPECIAL PROGRAMS

4.3.2 Introduction to Integrated Case Management: Leprosy, Buruli Ulcer, Elephantiasis, Hydrocele and Yaws

**TIME:** 6 hours, 45 minutes
**OBJECTIVES**<br><br>**By the end of the lesson, participants will be able to:**<br>• Describe what Leprosy, Buruli Ulcer, Elephantiasis, Hydrocele and Yaws are and how they are spread<br>• Explain how Leprosy, Buruli Ulcer, Elephantiasis, Hydrocele and Yaws are managed<br>• Describe how Leprosy, Buruli Ulcer, Elephantiasis, Hydrocele and Yaws can be prevented<br>• Provide psychosocial support and link patients to peer advocates<br>• Deliver messages to reduce stigma and discrimination for people with Leprosy, Buruli Ulcer, Elephantiasis, Hydrocele and Yaws<br>• Train affected persons with NTDs who require home-based self-care<br>• Identify, refer, report and follow up NTDs cases<br>• Correctly complete the routine visit form, patient tracing form and community alert and referral form.

Neglected Tropical Diseases (NTDs) are different groups of infectious diseases that are present in tropical countries all over the world. They affect more than one billion people. Leprosy, Buruli Ulcer (BU), Elephantiasis (EL), Hydrocele (H) and

Yaws (YA) are five examples of NTDs that affect people in Liberia. These infectious diseases develop very slowly and are caused by germs that affect the skin, hands, feet, eyes, etc. Any person regardless of age, sex, class can contract these diseases. Although these NTDs continue to be a public health problem in Liberia, the good news is that leprosy, Buruli Ulcer, Elephantiasis, Hydrocele and Yaws can be managed with effective treatment!

The aim of this session on Integrated Case Management (CM) is to give participants general information about leprosy, Buruli Ulcers, Elephantiasis, Hydrocele and Yaws.

LESSON 4.3.3

Leprosy, Buruli Ulcer (BU), Elephantiasis (EL), Hydrocele (H) and Yaws Identification, Referral and Management

4.3.3.1 LEPROSY

<table>
<tr><th colspan="3">WHAT IS LEPROSY?</th></tr>
<tr><td colspan="3">• Leprosy is an infectious disease that someone can get through the air and it develops very slowly.
• Leprosy is caused by a germ.</td></tr>
<tr><td>Loss of feeling 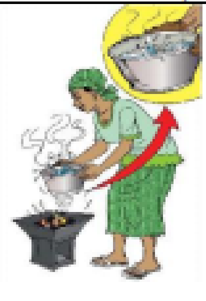</td><td>Loss of eyebrows, thick or lumpy earlobes, thick Nerves 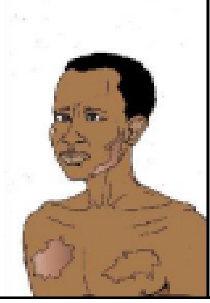</td><td>Deformities of hands and feet 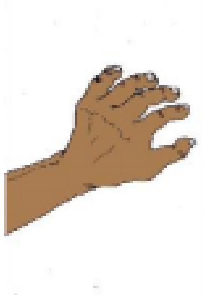</td></tr>
</table>

HOW IS LEPROSY SPREAD?	
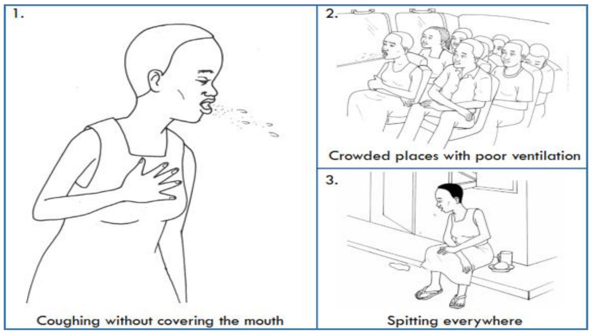	• Leprosy germs are passed through the coughing and sneezing of a person with Leprosy.<br>• In most cases, leprosy germs are spread through long-term contact with a person who has the disease and who has not been given effective medicine.

FORMS OF LEPROSY	
Non-Infectious (Can't spread)	
• Up to 5 patches<br>• Cannot pass Leprosy to others	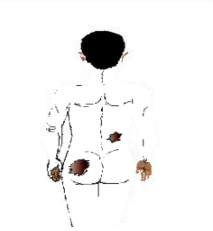 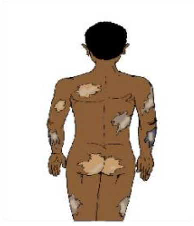 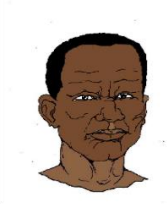
Infectious (Can Spread)	
• 6 or more patches Can pass Leprosy to others (until treatment starts)	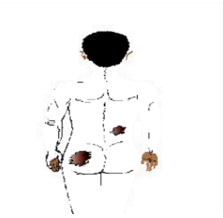 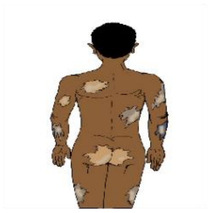 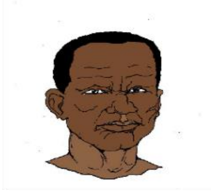

SIGNS AND SYMPTOMS OF LEPROSY

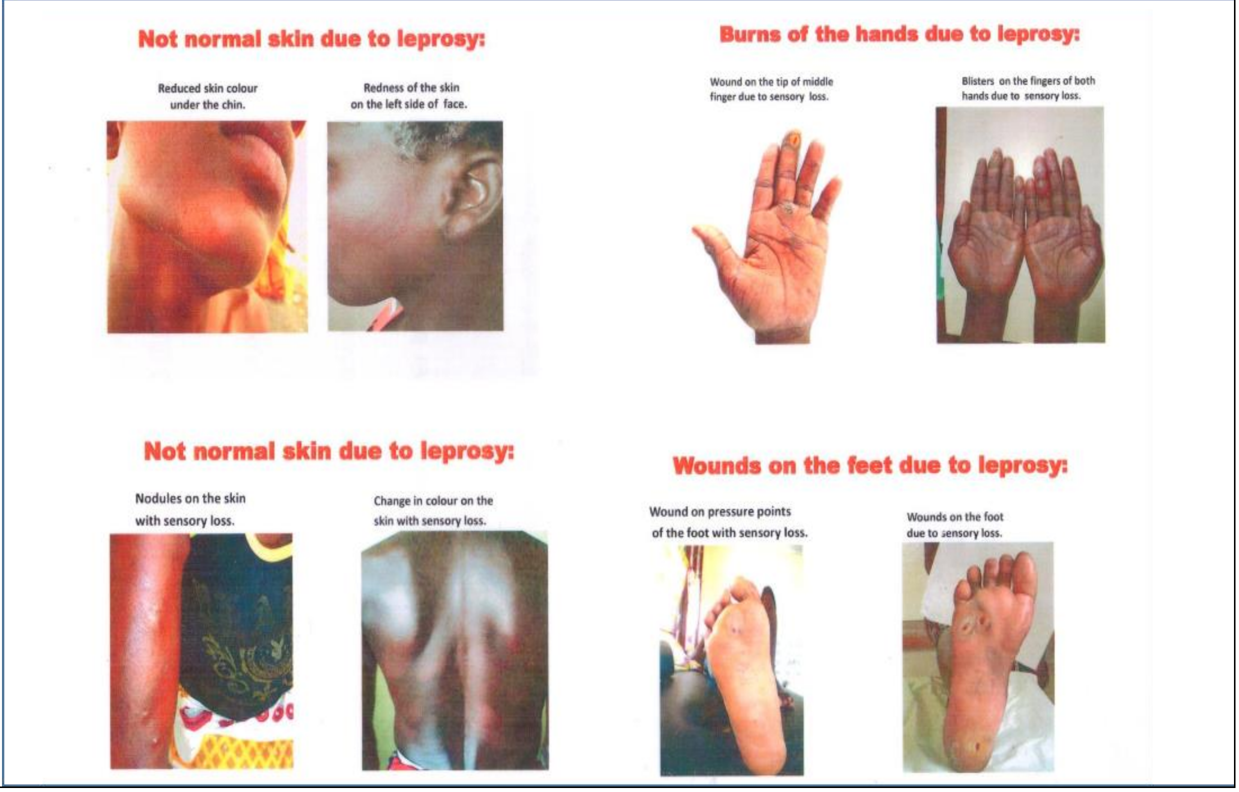

HOW IS LEPROSY MANAGED?

Educate the patient about the following:

- Leprosy can be treated with medicine provided by the health facility.
- Complete the medicine provided by the clinic within the time given.
- Soak the affected hands and feet in a bucket of salt water for about 15 minutes.
- Wrap grease or oil on the affected hands and feet.
- Wear soft shoes that protect the feet from being hurt.
- Wear soft gloves when doing works that could hurt the hands such as brushing, beating pepper and holding hot pots.
- Check yourself every morning and evening to see if you have any sore.

RECOGNIZING SERIOUS SIDE EFFECTS OF LEPROSY

Serious side effects of leprosy medicine:

Educate the patient to look out for the following serious signs:

- Rash (Craw-craw)
- Jaundice (Yellow jaundice or yellow eyes)
- Lack of appetite
- Nausea (feel like vomiting)
- Vomiting
- Flu-like symptoms (chills, fever, headache, bone pains, weakness)
- Big change in mood (very excited or very frightened)

(Whenever a patient has any serious sign, carry patient to the health facility immediately!)

RECOGNIZING NOT SERIOUS SIDE EFFECTS OF LEPROSY

Educate the patient that the following “not serious” signs and symptoms can happen to some patients when they are taking Leprosy medicine:

- Urine is slightly red
- Skin turns brownish-black and shows dryness over the course of months
- Tiredness / Fatigue
- Hunger

(If a patient has any of these signs, he or she should go to the health facility within one week!)

HOW TO PREVENT LEPROSY AND IT'S DISABILITIES								
Covering mouth and nose when coughing and sneezing	+	Early screening and identification of patches	+	Early confirmation of Leprosy	+	Early treatment	+	Good drug adherence
= Prevention of damage and disability and GOOD TREATMENT OUTCOME								

ROLE PLAY
A CHA/CHV is invited by the Community Health Committee to come and give a health talk about Leprosy. After the CHA/CHV gives the health talk the chief asks the CHA/CHV to come back to his house to talk to his wife about leprosy because she couldn't be present at the group health talk. She was washing and caring for her children at the house.<br><br>The CHA/CHV arrives at the chief's house and the wife of the chief welcomes her inside and asks her to sit down.<br><br>The CHA/CHV gets out her job aid and begins to give a health talk to the wife and her children on what leprosy is, how it is spread, some signs and symptoms.

4.3.3.2 BURULI ULCER

WHAT IS BURULI ULCER?
• Buruli ulcer is disease that can make knot, swelling or hardness on the skin. It can lead to (make) sore when the disease gets worse.<br>• This is also known as "ever-lasting sore."

FORMS OF BURULI ULCER
Buruli Ulcer can appear in five different ways or forms

<table>
<tr><td>Nodular (Knot) Form
Small, hard, painless knot or swelling under skin.</td><td>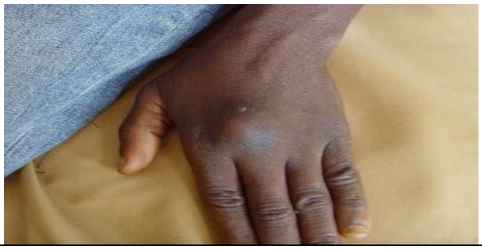</td></tr>
<tr><td>Plaque (Hard skin) Form
Plaque form of BU appears as a large, hard and painless part of the skin that is more than 1 inch wide.</td><td></td></tr>
<tr><td>Edema (Swelling) Form
Edema form of BU is a large, painless swelling of the skin that is usually seen on one arm, leg, face or any part of body.</td><td></td></tr>
<tr><td>Ulcer (Sore) Form
-Ulcer form is generally not painful; it usually has loose edges.</td><td>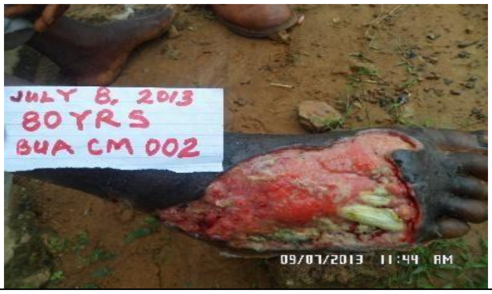
</td></tr>
<tr><td>Osteomyelitis (Bone) Form
-Ulcer may appear as small hole in the skin that goes toward the bone. The hole usually produces puss and is painful.</td><td>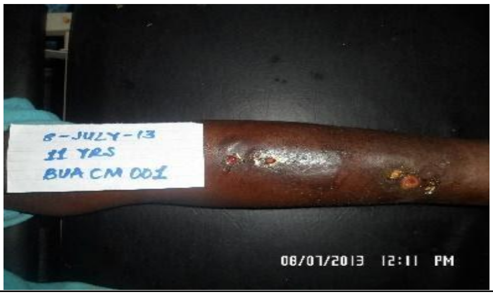
</td></tr>
</table>

How Can Buruli Ulcer be Managed?

- Buruli ulcer can be cured by taking the medicine on time.
- Also patients with sore will need to visit the health facility frequently for sore dressing.

Side Effects of Buruli Ulcer Medicine

Educate the patient about the following side effects of Buruli Ulcer medicines:

- Buruli ulcer medicine can affect some people in the following ways:

Serious side effects

- Yellow eyes (Yellow Jaundice)
- You cannot urinate good

(Whenever a patient has any serious sign, carry patient to the health facility immediately!)

Not serious side effects

- Feel like vomiting and no appetite
- Stomach pain
- Hunger

(If a patient has any of these signs, he or she should go to the health facility within one week!)

How to Prevent Disability

- Regular movement and exercise of the affected part of the body
- Participation in wound care involving family members
- Visit facility for wound dressing

4.3.3.3 Elephantiasis (Lymphedema or Big foot)

What is Elephantiasis?

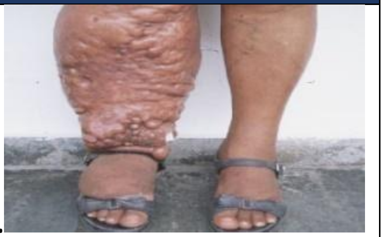

- It is a sickness that make people foot to get big.
- It can make people to feel very serious pains and have fever.

What Causes Elephantiasis?

- Elephantiasis is caused by a germ spread to humans through mosquitoes' bite.
- It is not caused by witch or country medicine.

How Can Elephantiasis be Managed?

It can be managed by doing the following:

- Taking Medicine (MDA) regularly for up to 8 years, to kill the germs.
- Practicing some self-help methods called Home based self-care (HBSC).
- The methods for practicing home based self-care are commonly called by a short cut called "C.A.R.E.".

You can manage Elephantiasis and prevent it from getting worse by using the CARE method:

- **Clean** & carefully wash the swollen area with soap and water every day
- **Apply,** disinfect any wounds by using cream that can kill both bacteria (antibacterial) and fungus (antifungal) cream whenever necessary.
- **Raise** the swollen part.
- **Exercise** any swollen arm or leg to move the fluid and improve the lymph flow.

HOW TO PRACTICE HOME-BASE SELF-CARE	
**CLEAN**	• Wash the foot with clean water and soap everyday (don't use hot water)<br>• Use soft cloth and carefully wrap the soap between the toes and skin folds<br>• Rinse the foot with plenty of clean water (don't use hot water)<br>• Take time and dry foot very well using a clean towel. No force. No rubbing.<br>• Make sure you dry the skin between the toes and skin folds.
**APPLY**	• Apply (wrap) anti-fungal and antibacterial cream on any part of the foot with sore or skin disease, so that germs will not enter the foot.<br>• Make sure all skin sicknesses are clean and dried very well before wrapping the cream.<br>• Then use clean cloth and cover all the sores and areas with skin disease to stop flies.
**RAISE**	• Raise the foot up in the air anytime you are sitting down or sleeping.<br>• You can put small bench or pillow under the foot to lift the foot up.
**EXERCISE**	• Exercise the sick foot 5 to 15 times a day so that the pain can go away.<br>• The exercises can be done anywhere and at any time.<br>• While exercising, twist the joints in all directions. But don't scrip any sore or bump.
**FOOT WEAR**	• Wear soft slippers or scandal to protect the bottom of the foot from injury<br>• It is better to use sandals that are not tight and allow air to reach all around the foot.<br>• Make sure the scandal should not cause rubbing or blisters.<br>• If blisters develop, don't burst them. Just take care of them until they get well.

How Can we Prevent Elephantiasis?
• By sleeping under treated mosquito net.<br>• Taking part in Mass Drug Administration.<br>• By keeping the environment clean and avoid water setting in old cans or cups.<br>• By keeping windows and doors closed during evening hours after 5pm.

4.3.3.4 Hydrocele

What is Hydrocele?
• It is water in the scrotum (men private part) that causes swelling
**What Causes Hydrocele?**
• Hydrocele is caused by filarial germ spread to humans through mosquito bite.<br>• It cannot be spread from one person to another through direct contact.

How can hydrocele be managed?
• It can be treated by surgery or operation at the hospital.<br>• Take MDA (NTDs mass drug administration) for up to 8 years, to kill the germs.

How can we Prevent Hydrocele?

It can be treated by surgery or operation at the hospital.

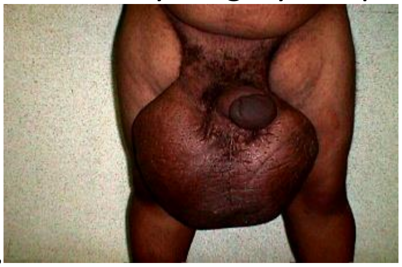

- It can be prevented by sleeping under treated mosquito net.
- Taking part in the Mass Drug Administration

- By keeping the environment clean and avoid water setting in old cans or cups
- By keeping windows and doors closed during evening hours after 5pm

4.3.3.5 YAWS

What is Yaws?

- Is a disease that affects the skin by making yellow bumps and sore
- It is more common in children less 15 years of age but can also affect adults. .

Forms of Yaws

There are 2 common forms of Yaws. They are Yaws Papule and Yaws Ulcer.

Yaws Papilloma (Yaws Bump) **Yaws Ulcer (Sore)**

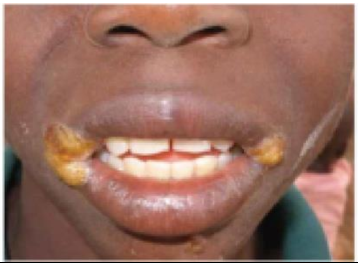

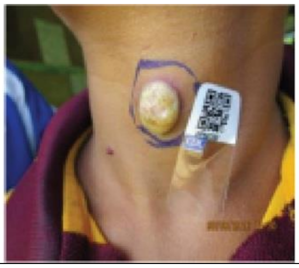

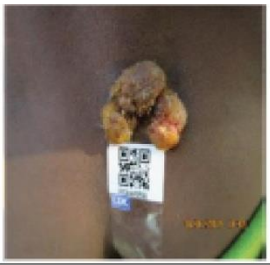

What Causes Yaws?

- It is caused by germs that spread from person to person through direct contact.
- It is not spread through the air or water.

How is Yaws Managed?

- It can be managed by taking the medicine on time.
- The health worker will give the patient some medicine to take on the spot, at the clinic. Once the person swallows the medicine on the spot, he or she will not need any other medicine again.

How can we Prevent Yaws?
• It can be prevented by:<br>○ Practicing good personal hygiene and avoid overcrowding<br>○ Refer to the health facility anybody you notice with strange bump or sore.

Lesson 4.4

Stigma and Discrimination

STIGMA AND DISCRIMINATION FOR NTDS

What is Stigma and Discrimination?
• Stigma is the negative thinking about someone condition<br>• Discrimination is the behavior of treating people different from others because of negative thinking about the person condition.<br>○ Discrimination can happen in health facilities which may prevent people from getting good treatment. Some family, friends and community can drive away people affected by NTDs. Some other people with NTDs face harsh treatment in school, work place and market.

How Stigma and Discrimination affect NTDs Care:
People affected by NTDs are pushed aside because of:<br><br>• Myths (wrong belief) and misinformation (lies) increase the stigma and discrimination surrounding NTDs.<br>○ What are examples of myths and misinformation about NTDs? *(allow a few responses)*<br>▪ *Answer: People think NTDs are the result of curses or witchcraft due to something the patient or their immediate family did*<br>▪ *People believe that leprosy cannot be healed*<br>▪ *People believe Buruli ulcer is everlasting and the sore can never go away*<br>Fear of NTDs<br>• *What are some examples of how people fear NTDs? (allow a few responses)*

- *Answer: People are afraid that they will contact leprosy if the interact with someone who has the disease*
- *People are afraid of contacting BU when they see patients with sores*

Forms of Stigma

Self-stigma/internalized stigma

Negative thinking about oneself can make an NTDs patient feel shame and blame others. Self-stigma can make NTDs patients to feel sorry for their condition and to not complete their treatment.

Healthcare stigma

Health workers can avoid touching patients with a sore, skin rashes or other NTDs signs and delay or deny treatment. Some health workers may demand additional payment for treating people with NTDs. Healthcare workers may violate a patient's privacy and confidentiality, including disclosure of a person's diagnosis to family members or hospital employees without permission.

Employment stigma

In the workplace, people affected by NTDs may suffer stigma from their co-workers and employers, such as avoiding the person and gossiping about the person.

Community and household level stigma

Community-level stigma and discrimination towards people affected by NTDs can force people to leave their home and change their daily activities.

How is Stigma Managed?

reate awareness through house to house education or large group health awareness about what NTDs are, how one can get NTDs, how it can be treated and how to prevent NTDs in the home/Community.

race lost-to-follow-up clients confidentially! Find and return patient that you have been linked with and has accepted you to support them in care and treatment

- **Support clients** by helping them to have support for their daily medications
- **Watch** for side effects of medications

- **Encourage treatment** completion and returning to the health facility for medication
- **ncourage nutritious food** that will keep them strong while they take their medication like nuts, fruit, meat and vegetables
- **Encourage client** to provide list of contacts for tracing (specifically: Yaws and Leprosy)
- **Link the client** to the health facility as needed
- **Remind the clients** that they can be healed if they complete their treatment
- **Complete forms** related to case management of NTDs

DECLARATION OF CONFIDENTIALITY

• Your job as a CHA/CHV is to understand stigma and discrimination, combat it and respond to it by doing the following:

- **Educate** your community about diseases that people who are discriminated against often have (neglected tropical diseases, and mental health concerns).
- **Correct** misunderstandings and myths about these diseases.
- **Encourage** community members to provide support to people living with these diseases
- **Be a role model** by respecting and caring for people living with these diseases
- **Recognize signs** of depression (being sad and staying down alone) and thoughts of suicide and seek the help of your supervisor or clinician.
- Above all, **KEEP ALL CLIENT INFORMATION CONFIDENTIAL.**

• To show your (CHAs/CHVs) support, you should take an oath together called a '**declaration of confidentiality'** to make sure you are all clear and in agreement on what it means to keep client information confidential.

I _____________________________________ declare on ___ (date) that I will keep the personal information of all clients assigned to me completely confidential. This means that:

- I will not share any client information (name, sex, location of house, phone number, patient records) with any friend, family member or any other person other than my supervisor
- I will not talk about any client information with any friends, family members, neighbors or anyone else in the community other than my supervisor
- I will not show any client forms (Patient Tracing Forms, Home Visit Forms, Patient Referral Forms, or treatment cards) to any friends, family members, neighbors or anyone else in the community other than my supervisor
- I will commit to keep all client information safe in my back bag or house and I will not allow any friends, family members or anyone else to go through my bag and look at my documents
- I will commit to respect my client's wishes for privacy and where he/she would like to meet for counseling.
- I will commit to doing everything in my power to keep all client information 100% confidential.

So, Help me God.

**CASE STUDY**: PREVENTING AND MANAGING STIGMA AND DISCRIMINATION
Victor is a CHA/CHV and has been working with a woman called Korto, who has infectious leprosy. She has been on treatment for a month and can no longer Spread the disease to another person. He recently found out that Korto has been abandoned by her family members when they found out she has leprosy. With Korto's permission, Victor has spoken with her family members and tried to help, but they will not take her back.<br>Questions:<br><br>1. Why do you think the family abandoned Korto?<br>2. What would you do in this situation?

3. How should such situations be prevented in the future?

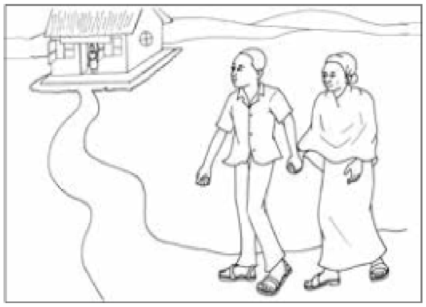

- Maintain patient privacy/secret.

- Help people with leprosy, BU, EL, Hydrocele and Yaws to complete their treatment by taking their medication every day.

- Support people living with NTDs to prevent further injury (help protect hands, feet, limbs and eyes.

- Inform a health worker of any side effects of medication or worsening of NTD symptoms.

Source: PIH Accompagnatuer Manual

LESSON 4.3.5 REFERRAL AND FOLLOW-UP PROTOCOLS

Case Referral

- Provide community health workers with case detection and referral tools
  - Reporting and referral forms
  - Community health ledgers.
- Step 1: Identify a person with skin condition like what is in the visual aid
- Step 2: Ask if the person is currently on treatment or have received treatment for this condition in the past
- Step 3: If the answer is no, fill in the referral form. If the answer is yes that the person is currently on treatment, ask to see the medicine. If the answer is yes that the person has completed treatment, ask the person to see the Leprosy Card If the person had Leprosy, and if the person is not a Leprosy patient, thank the person and end the conversation. Make sure you inform the CHSS to follow-up on the patient who say he or she completed treatment. .
- Step 4: Ask the person if he/she is able to go to the health facility

- Step 5: If the answer is yes, give the referral form to the person and ask the person when he/she will go to the facility so you can schedule your follow up visit.
- Step 6: If the person does not have the means to get to the health facility, continue to encourage the person to go to the health facility. Inform the CHSS.
- Step 7: If the person says that he/she cannot go to the health facility due to fear of treatment or some misinformation, provide counseling and link this person with a peer advocate.
- Step 8: After the introduction to the peer advocate, follow up with the person to make sure they visit the health facility with the referral form
- Step 9: Collect feedback forms from all persons referred

Feedback Protocol

- Within 24 hours of referring a person:
  - Step 1: visit the suspect's home to obtain feedback form
  - Step 2: If the suspect has not visited the health facility, continue visiting and encouraging the suspect to visit the health facility
  - Step 3: If the suspect does not go to the health facility after a week, then report the case to the CHSS

Follow up

When the person returns from the health facility within 24 hours:

- Step 1: visit the person home
- Step 2: Read the feedback form to determine diagnosis
- Step 3: If the person is diagnosed with L**eprosy or Yaws** you do the following:
  - Ask the patient to show you the medication and other supplies given by the health facility
  - Ask the patient if their family and friends know about their diagnosis
    - If the patient says yes: ask them to call the friend or family member that is aware of the condition to join the discussion
      - Discuss the treatment regimen with the patient and their family or friend present
      - Ask the family or friend present to help monitor if patient is taking their medicine according to schedule.

        - Ask the family or friend to help support the patient with food if the patient doesn't have enough food.
    - If the patient says he/she has not shared medical results with friends or family members then do the following:
        - Educate the patient on the benefits of family support
        - Share the consent form with the person to sign with a pen or thumb
  - Use the contact tracing form and list the names and contacts of family members living in the same house with the person
  - Those contacts that are present, show them the visual aid and ask if anyone have those conditions
  - If any of the contacts confirm to have any conditions in the visual aid, ask them to allow you to see the area
  - If you confirm that any of the contacts have similar conditions then use the referral protocol.
  - If the patient has a wound, ask for next scheduled visit to the health facility
  - Schedule follow up visits once a week with patient
  - Complete follow up form during each visit and report it to CHSS

- Step 3: If the patient is diagnosed with **elephantiasis**, do the following:
  - Ask the person to show you the self-care kit given by the health facility
  - Ask the patient if their family and friends know about their diagnosis
    - If the patient says yes: ask them to call the friend or family member that is aware of the condition to join the discussion
        - Discuss the self-care management with the patient and their family or friend present
        - Ask the family or friend present to help monitor if patient is practicing self-care practice according to training
    - If the patient says he/she has not shared medical results with friends or family members then do the following:
        - Educate the patient on the benefits of family support
        - Share the consent form with the person to sign with a pen or thumb

- Ask patient to visit health facility whenever they experience pain at the sight of the affected area or fever.
- If the patient has pain or fever, fill in the follow up form and ask them to visit the nearest health facility.
- Schedule your next visit

- Step 3: If the patient is diagnosed with **Buruli ulcer**, do the following:
  - Ask the person to show you the medication and other supplies given by the health facility
  - Ask the patient if their family and friends know about their diagnosis
    - If the patient says yes: ask them to call the friend or family member that is aware of the condition to join the discussion
      - Discuss the treatment regimen with the patient and their family or friend present
      - Ask the family or friend present to help monitor if patient is taking their medicine according to schedule.
    - If the patient says he/she has not shared medical results with friends or family members then do the following:
      - Educate the patient on the benefits of family support
      - Share the consent form with the person to sign with a pen or thumb
  - If family members living in the same house are present, show them the visual aid and ask if anyone have those conditions
  - If any of them confirm to have any conditions in the visual aid, ask them to allow you to see the area
  - If you confirm that any of the family members have similar conditions then use the referral protocol.
  - Schedule follow up visit three days to make sure patients visit health facility at least twice a week for wound dressing
  -
  - Step 3: If the patient is diagnosed with **hydrocele** do the following:
    - Ask patient if they have been scheduled for surgery
    - If the patient says no then ask them for their next follow up at the health facility
    - If the patient has a surgery date, plan a follow up visit for one week after surgery date. This will be the last follow up.

Lesson 4.3.6

Recording and Reporting Forms

Practice with Module 4.3 Essential Forms

Refer to Appendix

You can practice filling in each form within the appendix. Use the (scenarios) short stories below to fill in the forms.

Form Use Practice – 1: Filling the Routine Home Visit Form
Meet Grace. She is 31 years old and has two beautiful children. Grace tested positive for Buruli ulcer in January 2015. Grace was very motivated to start her medication and reports 100% adherence. You also see her pills and verify that she has been taking her pills correctly. She reports no side effects or discomfort. She reports sleeping and eating well and looks well on the outside also. You do not observe her take her medication each day, but she keeps record in a notebook in her kitchen.

Form Use Practice – 2: Filling the Patient Tracing Form
Meet Martha. She is 13 years old and she has leprosy. She has been taking her medicine for the last two months and her father makes sure she takes her medication every day. Last week, when you went to visit Martha's home, you couldn't find her.

Form Use Practice – 3: Filling the Contact Tracing Form
Mambu Kamara, a 36 year old male, was diagnosed with leprosy at the Tienii clinic, Tewoi district, Grand Cape Mount. He lives in Mampo village three hours walk away from the clinic. On the feedback form you received his patient ID number is 003 and his contact number is 07709834567. Mambu is married and lives with his wife (Helen Kamara, 35 years old) and two children (Massa Kamara 16 yr old and Abu Kamara 17 year old). He also has his mother and father in laws (Satta Varney 65 year old and Soni Varney 70 year old) his sister two sons (Varney 20 year old and Kadii Sambulleh 17 year old). You have come

to conduct your first home visit with Mambu Kamara. Now fill in the contact tracing form during this visit.

Form Use Practice – 4: Filling the Referral and Counter Referral Form

Meet Wolobah Joemah. He is a 12-year-old boy who farms for a living in Betibah Town, Voinjama District, Lofa County. He reports being very healthy, eats well, does not drink or smoke. Recently, he had visited his younger brother and family in another county. Bernard came back with sore mouth. He reports having the sore for over two weeks. You learned about his sore mouth when you visited him this morning. You saw yellow bumps at the corner of his mouth.

Form Use Practice – 5: Filling the Referral and Counter Referral Form

You are a CHA/CHV and it is the end of the Month and your supervisor asks you to submit your activities for the month on the CM NTDs services you've provided to the community for the month.

You have a stack of 10 NTDs Contact tracing Forms (3 LTFU Buruli ulcer patients, 5 Default leprosy patients), you have a stack of 6 Home Visit forms (2 elephantiasis patients, 2 hydrocele patients, 2 yaws patients) and you completed 12 referral forms at a Health Talk during Saturday Awareness. Those are the only referrals you have made this month.

Tally all these numbers of your form and submit to your supervisor.

Form Use Practice – 6: Home Visit Form

Mr. James, 24 years was a teacher, He stopped going to school to work, become silent, not interested in his life. He developed swelling in his scrotum (man private part). You must show pictures of swelling and discuss the symptoms of this disease and how to help him.

Form Use Practice – 7: CM NTDs Contact Tracing Form

Mambu Kamara, a 36-year-old male, was diagnosed with leprosy at the Tienii Clinic, Tewoi District, Grand Cape Mount. He lives in Mampo Village which is three hours walk away from the clinic. On the feedback form you received, his Patient ID number is 01303 and his contact number is 07709834567. Mambu is married and lives with his wife (Helen Kamara, 35 years old) and two children (Massa Kamara 16 yr old and Abu Kamara 17 yr old). He also has other family members living with him in the same house, including his mother and father in

laws (Satta Varney 65 yr old and and Soni Varney 70 yr old), his sister's two sons (Varney 20 yr old and Kadii Sambulleh 17 yr old). You have come to conduct your first home visit with Mambu Kamara. Now fill in the contact tracing form during this visit.
