# Supplementary material for: The development, implementation, and evaluation of an optimal model for the case detection, referral, and case management of Neglected Tropical Diseases
Source: PLoS One. 2023 May 10;18(5):e0283856. doi: 10.1371/journal.pone.0283856 (PMC10171595; doi:10.1371/journal.pone.0283856)
Supplement: S4 Appendix — (PDF) [file pone.0283856.s009.pdf]

1. **Routine Home Visit Form**

CHA/V Routine Visit Form

v.0.8

CHA/V Name:	CHA/V ID:	Comm. Names:	Comm. ID1
CHSS Name:	CHSS ID:		Comm. ID2
County:			Facility Name:

		Mod. 1								Mod. 2		Mod. 3			Mod. 4		
HHID	Visit Date (dd-mm-yyyy)	# of births	# of still births	# of neonatal deaths: 0 day - 1 mo	# of post-neonatal deaths: 1mo - 1yr	# of child deaths: 1-5 years	# of maternal deaths	# of community triggers		# of pregnant women	# of women 15 - 49 years	# of children under 5	# of sick children under 5	# of well children under 5	# of TB patient	# of ART patient	# of CM NTDs Patients
Total																	

2. Community Trigger and Referral form

1.3 Community Trigger & Referral Form

v.0.25

Section A Referral [Community → Facility] *to be triaged immediately*

The CHA/CHV fills this out, and submit to the Health facility (CHSS, OIC, SFP)

Patient Name:

Community:

District:

County

Sex: ◯Male ◯Female

Facility or POE:

Date (DD/MM/YYYY):

CHA/V Name:

Patient Age: ◯Years ◯Months

CHA/V Phone Number:

Crossed Int. Border in last 1 month ◯Y ◯N

IDSR-ID:
(Filled by Health Facility)

Priority Disease Triggers

① Acute flaccid paralysis (Polio)
② Acute watery diarrhea / Cholera (Runny stomach)
③ Bloody Diarrhea (pu-pu with blood)
④ Human Rabies (Dog bite)
⑤ Measles
⑥ Viral Hemorrhagic Fever (Ebola, Lassa Fever, & Yellow Fever)
◯ Other (write in):
⑦ Meningitis (Stiff neck)
⑧ Maternal Death (Big belly death)
⑨ Neonatal Tetanus (Jerking sickness)
⑩ Neonatal Death (Young baby death)
⑪ Unknown health problems grouped together
⑫ Any death in human or group of animals that you don't know why it happened

Core Referral CHA and or CHV Where Applicable

◯ Family Planning
◯ Mental Health
◯ Child Vaccination
◯ Elephantiasis
◯ Child Health
◯ Tuberculosis
◯ HIV
◯ Yaws
◯ Maternal & Infant Health
◯ Leprosy
◯ Buruli Ulcer
◯ Hydrocele

Case description & any danger sign observed

Describe any investigation or treatment

Facility Health Worker - Tear Here

Section B Counter-Referral [Facility → Community]

For the Facility Health Worker: He/she should tear at the dotted line above and return to the CHSS to take to the CHA/CHV

Patient Name:

CHA/CHV Name:

Date [DD/MM/YYYY]:

Community:

Facility Worker Name:

Health Facility:

Facility Worker Phone #:

Facility Worker Position:

Case Definition Met ◯Y ◯N

IDSR-ID:

Follow up plan & instructions to CHA/CHV:

Actions Taken (tick all that apply)
◯ Treated and sent home
◯ Placed in isolation unit
◯ Admitted ◯ Referred
◯ Sample collected
◯ Other (write in):

3.

INTGRATED NTDs CONTACT REGISTER

MINISTRY OF HEALTH, LIBERIA

PATIENT DETAILS

Client Name: ______________________ Sex: M/F Age: ___ (yrs) Phone: ______________ Current Village/Town: ____________

Disease Type: ______________ Hosp. ID: ______________ HF Name: ______________ HF County: ____________

CONTACTS DETAILS

#	FULL NAME	DATE OF BIRTH e.g.:15-Jan-2019	SEX (M/F)	SCREENING STATUS Partial/ Complete	SCREENING DATE e.g.: 15-Jan-2019	STATUS (Neg., Suspected Lepr. PB, Lepr. MB, Yaws, etc.	REMARKS

CONTACT TRACER DETAILS

Name: ______________________ Title: ______________ Phone: ______________ Sign & Date: ______________

Health Facility: ______________ Phone #: ______________ District: ______________ County: ______________

4.

v.0.7

4.0 CHA/V Monthly Service Report [p.2]

HIV, TB, Leprosy, Buruli Ulcer, Hydrocele, Elephantiasis, Yaws, Mental Health<br>B. Integrated Case Management of Neglected Tropical Diseases (CM NTDs)	W. 1	W. 2	W. 3	W. 4	Total
4.3A # of Patients referred to health facility for CM NTDs testing					
4.3B # of CM NTDs patients treated					
4.3C # of CM NTDs patients treated					
4.3D # of default CM NTDs patients treated					
4.3E # of new CM NTDs patients visited					
4.3F Total # of CM NTDs Patients visited					

C. HIV	W. 1	W. 2	W. 3	W. 4	Total
4.4A # of HIV patients referred to health facility for testing and service					
4.4B # of lost to follow-up ART patients traced					

D. Mental Health	W. 1	W. 2	W. 3	W. 4	Total
4.5 # of patients referred to health facility for Mental Health services					

5.
